# Mutational spectrum of adult T-ALL

**DOI:** 10.18632/oncotarget.2218

**Published:** 2014-07-15

**Authors:** Martin Neumann, Sebastian Vosberg, Cornelia Schlee, Sandra Heesch, Stefan Schwartz, Nicola Gökbuget, Dieter Hoelzer, Alexander Graf, Stefan Krebs, Isabelle Bartram, Helmut Blum, Monika Brüggemann, Jochen Hecht, Stefan K. Bohlander, Philipp A. Greif, Claudia D. Baldus

**Affiliations:** ^1^ Charité, Universitätsmedizin Berlin, Campus Benjamin Franklin, Department of Hematology and Oncology, Berlin, Germany; ^2^ Clinical Cooperative Group ‘Leukemia’, Helmholtz Zentrum München, German Research Center for Environmental Health, Munich, Germany; ^3^ Department of Internal Medicine 3, Ludwig-Maximilians-Universität (LMU), Munich, Germany; ^4^ Goethe University Hospital, Department of Medicine II, Hematology/Oncology, Frankfurt/M., Germany; ^5^ Laboratory for Functional Genome Analysis, Gene-Center, Ludwig-Maximilians-Universität (LMU), Munich, Germany; ^6^ University Hospital Kiel, Department of Hematology, University Hospital Schleswig-Holstein, Campus Kiel, Germany; ^7^ Berlin-Brandenburg Center for Regenerative Therapies (BCRT), Charité Universitätsmedizin Berlin, Berlin, Germany; ^8^ Department of Molecular Medicine and Pathology, The University of Auckland, Auckland, New Zealand; ^9^ German Cancer Consortium (DKTK), Heidelberg, Germany; ^10^ German Cancer Research Centre (DKFZ), Heidelberg, Germany

**Keywords:** Acute lymphoblastic leukemia, targeted therapy, T-ALL next generation sequencing, pathways, gene panel

## Abstract

Novel target discovery is warranted to improve treatment in adult T-cell acute lymphoblastic leukemia (T-ALL) patients. We provide a comprehensive study on mutations to enhance the understanding of therapeutic targets and studied 81 adult T-ALL patients. *NOTCH1* exhibitedthe highest mutation rate (53%). Mutation frequencies of *FBXW7* (10%), *WT1* (10%), *JAK3* (12%), *PHF6* (11%), and *BCL11B* (10%) were in line with previous reports. We identified recurrent alterations in transcription factors *DNM2*, and *RELN*, the WNT pathway associated cadherin *FAT1*, and in epigenetic regulators (*MLL2*, *EZH2*). Interestingly, we discovered novel recurrent mutations in the DNA repair complex member *HERC1*, in *NOTCH2*, and in the splicing factor *ZRSR2*. A frequently affected pathway was the JAK/STAT pathway (18%) and a significant proportion of T-ALL patients harboured mutations in epigenetic regulators (33%), both predominantly found in the unfavourable subgroup of early T-ALL. Importantly, adult T-ALL patients not only showed a highly heterogeneous mutational spectrum, but also variable subclonal allele frequencies implicated in therapy resistance and evolution of relapse. In conclusion, we provide novel insights in genetic alterations of signalling pathways (e.g. druggable by γ-secretase inhibitors, JAK inhibitors or EZH2 inhibitors), present in over 80% of all adult T-ALL patients, that could guide novel therapeutic approaches.

## INTRODUCTION

T-cell acute lymphoblastic leukemia (T-ALL) in adults represents a disease with an unfavorable outcome[1]. While the cure rate in pediatric T-ALL patients exceeds 70%, a similar rate in adults is only observed for patients in the favorable risk group of thymic T-ALL as defined by the expression of CD1a[2,3]. Patients within the immature immunophenotypic group of early T-ALL as well as patients of the mature T-ALL subtype show a significantly inferior outcome[1]. Although allogeneic stem cell transplantation (alloSCT) in first complete remission has led to an improved outcome e.g. in the context of the German Multicenter Acute Lymphoblastic Leukemia (GMALL) trials, further therapeutic improvements are urgently warranted, in particular for high risk patients. In B-cell precursor (BCP-) ALL molecularly directed therapies like Rituxumab[4], the bi-specific antibody Blinatumomab[5], or tyrosine kinase inhibitors in Ph+-ALL[6] are well established. In contrast, targeted therapies are not available for T-ALL, with the exception of Nelarabine[7]. In order to identify targets for specific treatment strategies, a better understanding of the molecular background of T-ALL is necessary[8].

Previous to next generation sequencing (NGS), genetic alterations of leukemic blasts were mainly examined by cytogenetics to detect chromosomal rearrangements. In addition, immunophenotypic characterization, gene expression arrays, and copy number alterations were also used to categorize T-ALL[9]. Variations on a single nucleotide level had only been described for very few genes. One of the most relevant and frequent alterations are mutations of *NOTCH1* gene occurring in about 60% of all T-ALL cases[10-12]. In addition, mutations of *FBXW7*, another player in the NOTCH pathway, as well as mutations of *WT1* and *PTEN* were previously described[13-18].

Through NGS, molecular classification of T-ALL has dramatically expanded. Recurrent mutations in T-ALL affect genes involved in transcriptional processes (*BCL11B*[19], *RUNX1*[20], *GATA3*[21]), epigenetic regulation (*DNMT3A*[20,22], members of polycomb repressor complex (PRC2)[21]), JAK/STAT signalling[21,23-25] (*JAK1*/2/3, *IL7R*), ribosomal processes (*RPL10*, *RPL5)*[25], and various other functions (e.g. *WT1*[26], *CNOT3*[25], *PH[[27], MEF2C[28,29]*, *LEF1*[30]). These data, predominantly derived from pediatric T-ALL, suggest a highly heterogeneous and complex molecular background of T-ALL. While some of these alterations imply prognostic significance, comprehensive studies with focus on therapeutic targets in larger series of adult T-ALL patients are missing. This is of particular importance as most cancer genes occur at intermediate frequencies of 2-20% or even lower[31].

Thus far, molecular subgroups in T-ALL were defined mainly based on gene expression profile (GEP)[28,32,33] or immunophenotype[2,3]. Both classifications, based on GEP or immunophenotype reflect the physiological T-cell stage, in which growth arrest and malignant transformation occured[34]. The gene signature of the early T-cell precursor (ETP)-ALL reflects the expression profile of early thymocyte progenitors in the double negative (DN)1 stage and ETP-ALL also shows a distinct immunophenotype[35,36]. Importantly, ETP-ALL, which recently gained interest as it represents a subgroup of T-ALL with stem cell and myeloid characteristics[21,36-38], may serve as model for the design of novel molecular therapies. Although the classification of ETP-ALL based on gene expression and immunophenotype were only partly overlapping[29], the subgroup of ETP-ALL is already an ideal model for therapy approaches adapted to its distinct molecular characteristics. Specifically mutated genes, mainly affected in ETP-ALL include members of the (PRC2) or genes reflecting the stem cell and myeloid character of ETP-ALL like *FLT3, DNMT3A* or *KRAS.* This mutation pattern of ETP-ALL opens up potential options for targeted therapies[21,22]. This might be of special interest in a minimal residual disease (MRD) setting as a bridging therapy to alloSCT.

Whereas a number of putative driver mutations have been characterized, the spectrum of recurring alterations in larger cohorts and their relevance in different T-ALL subgroups remains unexplored. To unravel this spectrum and to explore potential targets for novel therapeutic interventions, we performed targeted high throughput sequencing of 88 candidate genes in 81 T-ALL samples of adult patients.

## RESULTS

### Single nucleotide variations and short indels in adult T-ALL

We obtained an average of 1.2 million reads for each sample resulting in an average coverage of 120 reads for the target region. Eighty percent of the targeted region was covered with a minimum of 20 reads (Supplementary Table S1). After exclusion of polymorphisms annotated in dbSNP135, 473 single nucleotide variations (SNVs) and short indels were identified with a minimum call of 20 reads, 313 of those resulted in changes in the coding sequence of the target region. On average, three (3.2) genes per patient were mutated, and 64 (73%) of the 88 genes were mutated in at least one patient (Supplementary Table S2). We identified three patients without any SNVs in the selected genes. One patient showed an aberrantly high rate of SNVs with 21/88 genes being mutated (Supplementary Table S3). The number of mutations in the selected genes did not correlate with the patients' age.

### Mutational spectrum of candidate genes in T-ALL

In total, fifteen of the 88 investigated genes were mutated in more than 5% of patients with nine genes showing a mutation frequency of ≥10%. As expected, the highest mutation rate with 53% was found for *NOTCH1*. Mutation frequencies of *FBXW7* (10%), *WT1* (10%), *JAK3* (12%), and *BCL11B* (10%) were in the range of previously reported frequencies[21,26,39,40]. Recently identified recurrent alterations in *DNM2* (17%), *PHF6* (11%), *DNMT3A* (5%) or *RELN* (5%) were confirmed in this larger cohort of adult T-ALL patients[20,21,27] (Table 2). Interestingly, genes involved in epigenetic functions such as *TET2* (5%), *SUZ12* (5%), *EP300* (5%) as well as genes that possess transcriptional activity like *RUNX1* (9%), *PTEN* (8%), *CBL* (5%), or *BCOR* (4%) were mutated in the range of 5-10% of our T-ALL patients.

Genes previously linked to ETP-ALL were also found to be mutated in the remaining non-ETP T-ALL subgroups including recurring mutations in the histone methyl-transferase *MLL2* (11%), frequently mutated in B-cell lymphomas[41-43]. Like in B-cell lymphoma, *MLL2* mutations were distributed over the entire gene locus without pointing towards a hot-spot region (Supplementary Figure S1). Similarly, the protocadherins *FAT1* (15%) and *FAT3* (12%) were altered not only in early T-ALL (*FAT1* 23%, *FAT3* 15%), but were also recurrently mutated - though in a lower frequency - in thymic T-ALL (*FAT1* 15%, *FAT3* 13%; Table 1).

**Figure 1:**
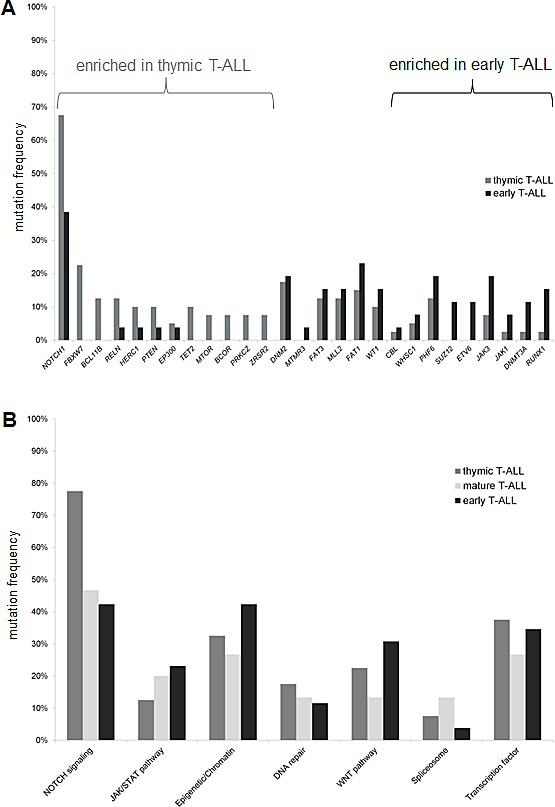
Comparison of mutation frequencies between the different T-ALL subgroups (A) Distribution for single genes and (B) according to the related pathways. Only genes with a mutation rate higher than 3% are shown.

Mutation frequencies for distinct genes appeared to significantly different across T-ALL subgroups (Figure 1a). This was most remarkable for members of the NOTCH pathway: *NOTCH1* showed a higher frequency in thymic (67.5%) compared to early T-ALL (38.4%, P=0.02). Consistent with this mature immunophenotype, *NOTCH1* mutation status was significantly linked to a clonal TCR rearrangement (64% clonal TCR rearrangement in *NOTCH1*mut vs. 36% in *NOTCH1*wt, P=0.01). *FBXW7* mutations, similar to *NOTCH1* mutations, occurred exclusively in the subgroup of thymic T-ALL (Table 1). Additional mutations exclusively found in the subgroup of thymic T-ALL included *BCL11B*, *TET2*, *MTOR*, *BCOR*, and *ZSRS2*. In contrast, genes of the JAK/STAT pathway (*JAK1*, *JAK3*) and the PRC2 complex (*EZH2*, *SUZ12*) as well as the transcription factors *ETV6* and *RUNX1* were predominantly mutated in the immature T-ALL subgroup (Figure 1a).

In addition, we found novel mutations in genes which, to our knowledge, have not yet been reported in T-ALL. Among these *HERC1,* functionally involved in DNA repair, was among the most frequently mutated genes. Other recurrently affected genes included the splicing gene *ZRSR2*, or *PRKCZ*, a gene also involved in DNA repair (Supplementary Table S3).

Overall, there was no obvious association between the mutation status of different genes. Some of the genes with low mutation rates occurred exclusively, including genes with redundant functions like e.g. the histone methyltransferases, *WHSC1* and *MLL2*. *WHSC1* (also known as *NSD2* or *MMSET2*) is associated with the prognostic unfavourable t(4;14) subgroup in multiple myeloma[44] and only very recently described in T-ALL[45,46]. We found *WHSC1* to be mutated in 6% of the patients in our cohort. When combining *WHSC1* and *MLL2* mutated cases, 17% of all patients revealed alterations of histone methyltransferase genes.

**Table 1:** Mutational spectrum and comparison of T-ALL subgroups Genes with mutations detected in at least 3% of the examined samples are shown. In parentheses are the percentages for each subgroup

	Mutational spectrum			
		T-ALL subgroups		
	total	thymic	mature	early
n	81	40	15	26
NOTCH1	43 (53.1%)	27 (67.5%)	6 (40.0%)	10 (38.4%)
DNM2	14 (17.3%)	7 (17.5%)	2 (13.3%)	5 (19.2%)
FAT1	13 (16.0%)	6 (15.0%)	1 (6.7%)	6 (23.0%)
FAT3	11 (13.6%)	5 (12.5%)	2 (13.3%)	4 (15.3%)
JAK3	11 (13.6%)	3 (7.5%)	3 (20.0%)	5 (19.2%)
PHF6	11 (13.6%)	5 (12.5%)	1 (6.7%)	5 (19.2%)
MLL2	10 (12.3%)	5 (12.5%)	1 (6.7%)	4 (15.3%)
FBXW7	9 (11.1%)	9 (22.5%)	0 (0.0%)	0 (0.0%)
WT1	8 (9.9%)	4 (10.0%)	0 (0.0%)	4 (15.3%)
BCL11B	7 (8.6%)	5 (12.5%)	2 (13.3%)	0 (0.0%)
HERC1	7 (8.6%)	4 (10.0%)	2 (13.3%)	1 (3.8%)
RELN	7 (8.6%)	5 (12.5%)	1 (6.7%)	1 (3.8%)
RUNX1	7 (8.6%)	1 (2.5%)	2 (13.3%)	4 (15.3%)
PTEN	6 (7.4%)	4 (10.0%)	1 (6.7%)	1 (3.8%)
DNMT3A	5 (6.2%)	1 (2.5%)	1 (6.7%)	3 (11.5%)
CBL	4 (4.9%)	1 (2.5%)	2 (13.3%)	1 (3.8%)
EP300	4 (4.9%)	2 (5.0%)	1 (6.7%)	1 (3.8%)
JAK1	4 (4.9%)	1 (2.5%)	1 (6.7%)	2 (7.6%)
MTOR	4 (4.9%)	3 (7.5%)	1 (6.7%)	0 (0.0%)
SUZ12	4 (4.9%)	0 (0.0%)	1 (6.7%)	3 (11.5%)
TET2	4 (4.9%)	4 (10.0%)	0 (0.0%)	0 (0.0%)
WHSC1	4 (4.9%)	2 (5.0%)	0 (0.0%)	2 (7.6%)
BCOR	3 (3.7%)	3 (7.5%)	0 (0.0%)	0 (0.0%)
ETV6	3 (3.7%)	0 (0.0%)	0 (0.0%)	3 (11.5%)
MTMR3	3 (3.7%)	0 (0.0%)	2 (13.3%)	1 (3.8%)
PRKCZ	3 (3.7%)	3 (7.5%)	0 (0.0%)	0 (0.0%)
ZRSR2	3 (3.7%)	3 (7.5%)	0 (0.0%)	0 (0.0%)

### Affected pathways and association with T-ALL subgroups

To address the complexity of this heterogeneous mutational spectrum, we focused on pathways with potential targets. In this study, the NOTCH pathway was affected in about 60% of all T-ALL patients (Figure 1B), including mutations in *NOTCH1* and *FBXW7* as well as in *NOTCH2*, *NOTCH3*, *HES1*, *JAG1*, and *JAG*2 (Supplementary Table S3). Mutations involving the *NOTCH* pathway were predominant in the thymic subgroup (75%) as compared to the early T-ALL (33%, P=0.004) subgroup. The spectrum of additional mutations between *NOTCH1* mutated and *NOTCH1* wildtype patients was not significantly different.

**Figure 2:**
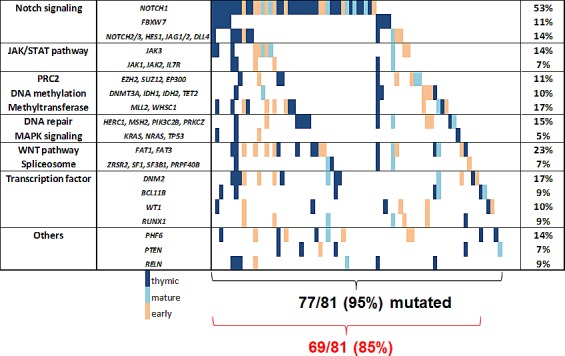
Mutational landscape of adult T-ALL In the right column mutations rates are shown for groups with functional similarity. The red brackets summarize pathways representing potential therapeutic targets and their frequency. Genes with a mutation rate below 5% are grouped with functional similar genes or are not shown.

Interestingly, over 35% of our T-ALL patients carried lesions in epigenetic modulators. Whereas DNA methylation modifiers (like *DNMT3A*, *TET2*, *IDH1, IDH*2) were affected in 9% of all cases, histone modifiers were even more frequently altered, including members of the PRC such as *SUZ12*, *EZH2*, or *EP300* and the histone methyltransferases *MLL2* and *WHSC1* (28%, Figure 2). Interestingly, chromatin modifying genes were slightly more frequently mutated in early compared to thymic T-ALL (42% vs. 32%, n.s.; Figure 1B).

The JAK/STAT pathway is of particular interest for the design of targeted therapies with the emergence of JAK inhibitors. Mutations in *JAK1*, *JAK2*, *JAK3*, *IL7R* occurred in 19% of all T-ALL patients, but these preferentially occurred in immature, high risk T-ALL cases. Among those, *JAK3* mutations were frequent (14%) and preferentially found in the early (19%) and mature (20%) subgroups compared to thymic T-ALL (8%, n.s., Table 1, Figure 1 and 2).

Another pathway of interest is the WNT pathway with a high rate of mutations in *FAT1* and *FAT3,* which is frequently altered in the immature T-ALL subgroups (Figure 2). The mutation frequency of *LEF1*, a main player in the WNT pathway, was unexpectedly low (1%), which may be due to the fact that larger deletions could be missed with our NGS approach.

Spliceosome mutations, described for myeloid and mature lymphoid malignancies, were present only in a minority (7.4%) of T-ALL (Figure 1B). Overall, pathways with a potential targeted treatment option were affected in 85% of all T-ALL patients. These included the NOTCH pathway, JAK/STAT pathway, WNT pathway, DNA methylation, chromatin modifying enzymes, spliceosome, and MAPK pathway (Figure 2).

### Variable allele frequencies suggest subclonal mutations

To identify mutations that may originate from the founding clone, we analysed the variant allele frequencies (VAFs) of all SNVs. In our cohort, T-ALL samples showed a wide spectrum of VAFs. For a founding clone, VAFs would be expected to be 44% (+/−7%)[47]. Within this T-ALL cohort, samples differed not only in the number of mutated genes, but also in range of VAFs for targeted genes.

**Figure 3:**
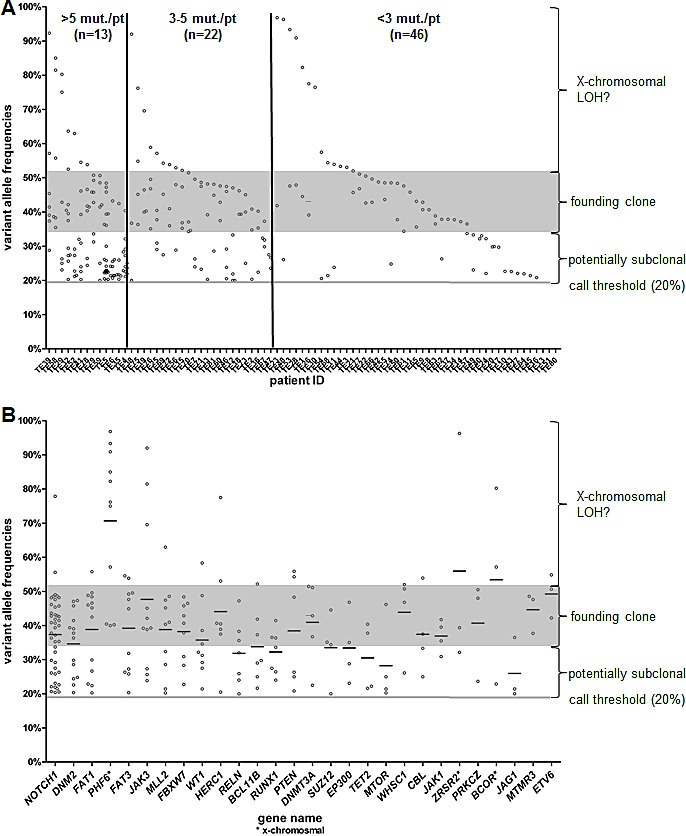
Variant allele frequencies (VAFs) of each individual patient (A) and each gene (B) are shown The grey shaded zone displays the expected range for a VAF in the founding clone.

The number of mutated genes varied widely across different patients (0-21/patient). The group of patients (n=13) with more than five mutated genes included cases with the majority of mutated genes linked to the founding clone. However, three cases had only one gene with VAFs of greater than 40% and the VAFs of the remaining genes were below 30%, pointing towards a subclonal structure of the leukemia. In patients with three or more mutated genes (n=36), we found at least one gene with VAFs in the range of a founding clone. On the other hand, we found 16 (18%) samples without any alteration with a VAF in the range of a founding clone (Figure 3A). These were all patients with two or less mutated genes and it is likely that the driver mutation was missed due to the gene selection.

The ideal target for an individualized treatment approach would be a driver mutation with a VAF in a founding clone. Although some of the genes with a mutation rate over 5% had predominantly VAFs over 40%, e.g. *NOTCH1* or *FAT1*, none of those could be exclusively assigned as founding clone. Interestingly, *NOTCH1* regarded as a prominent driver in T-ALL was found mutated on a subclonal level in 16 patients (37% of all *NOTCH1* mutated patients). Furthermore, in three patients *NOTCH1* showed at least two different alterations (Supplementary Figure S1). In all these cases one mutation had a VAF in the range of a founding clone, but the second mutation appeared to be present only within a subclone with a VAF below 30%.

Most of the recurrently mutated genes could be assigned to a founding clone in at least one patient. Some genes only showed low VAFs like e.g. *ABL1*, *FLT3*, *NRAS* or *SUZ12* and thus are presumably later events in leukemogenesis. All of these genes were mutated in only three or less T-ALL patients in our cohort. Among the genes with a VAF higher than 50%, *PHF6*, *BCOR*, and *ZRSR2* are located on the X chromosome (Figure 3B). Taken together, the spectrum of VAFs in T-ALL shows a highly heterogeneous pattern with none of the frequent (>10%) lesions being exclusively present in the founding clone.

## DISCUSSION

Although risk stratification and subsequent therapy intensification have led to an improved outcome in adult T-ALL, the cure rate of approximately 50% remains unsatisfactory. Unlike in BCP-ALL with established targeted therapies (Rituximab, TKI, potentially Blinatumomab), no targeted therapy is yet available in T-ALL. Therefore, molecular targets and implementation of individualized treatment options are sorely needed.

In our study, we investigated the mutational spectrum of a large adult T-ALL cohort to identify potential molecular targets. As previously reported, T-ALL shows, despite of common features regarding immunophenotype or gene expression, a highly heterogeneous mutational background[21,25]. However, most of the previously published data are generated in pediatric T-ALL patients. Here, we have investigated in an original and comprehensive study, a large set of candidate genes in a large cohort of adult T-ALL patients. This approach would allow us to identify also recurrent candidate genes altered in lower frequencies[31]. We were able to confirm a broad spectrum of mutations and this provides confidence that the detection of genetic lesions is accurately displayed in sequencing libraries and targeted NGS.

Among the most obvious mutations, we found genetic alterations in over 50% of the patients for *NOTCH1,* one of the best described events in T-ALL. All other reported recurrent mutations (among others *PTEN*, *PHF6*, *BCL11B*, or *WT1*) occurred in less than 20% of adult T-ALL patients[33]. The frequency of *NOTCH1* mutations as well as mutation rates for other well established genes like *WT1*, *FBXW7*, or *BCL11B* were in the range of previously reported incidences[33]. Another frequent alteration, genomic deletion of *CDKN2A*, was, however, not covered by our approach.

We also confirmed recurrent mutations in *DNM2*, *PHF6*, *PTEN*, *JAK3*, and *RUNX1*, which were only very recently discovered. The cadherins *FAT1* and *FAT3,* mutated in ETP-ALL[22], have not yet been described in non-ETP T-ALL of adults and were identified by our approach to be recurrently mutated across all subgroups of adult T-ALL. *FAT1* and its mutational inactivation have been linked to activation of the WNT pathway in solid tumors and to chemoresistance in chronic lymphocytic leukemia[48,49] and could serve as an attractive therapeutic target.

Furthermore, we found a high rate of mutations in *MLL2*, a histone methyltransferase, frequently mutated in various types of B-cell lymphomas[41-43]. Like in B-cell lymphomas, *MLL2* mutations were distributed over the entire gene without any obvious hot-spot region[41,50]. Interestingly, another histone methyltransferase, *WHSC1 (*also known as *MMSET/NSD2),* was recurrently mutated in T-ALL and, although in a small number of patients, mutually exclusive within *MLL2*. *WHSC1,* associated with the so called Wolf-Hirschhorn syndrome[51], was only very recently found to be mutated in pediatric ALL, particularly in t(12;21) ETV6-RUNX1 ALL[45,46], as well as in mantle cell lymphoma[42]. These results together with mutations in the PRC2 complex and in genes involved in DNA methylation unravel a yet unreported high frequency (of over 25%) of alterations in epigenetic regulators in adult T-ALL. This is in line with other hematologic malignancies like acute myeloid leukemia (AML),myelodysplastic syndrome (MDS) or diffuse large cell lymphoma[41,52,53]. These findings suggest that a very tight regulation of chromatin remodelling, especially for methylation of lysine 27 on histone H3, is required in physiological cell development and correct hematopoietic differentiation.

Interestingly, patients with an immature T-ALL immunophenotype showed a particular high frequency for mutations in epigenetic regulators and thus emphasize the similarity with myeloid malignancies. This is especially striking in the subgroup of ETP-ALL as already described by Zhang and colleagues[21]. We were unable to confirm the high mutation rate in the PRC2 members described for pediatric patients, but we frequently found mutations in regulators of DNA methylation, possibly related to preexisting lesions in hematopoietic progenitors in the elderly[22,54]. Taken together, the high frequency of mutations in epigenetic regulators offers new insights and potential therapeutic applications e.g. of EZH2 inhibitors, histone deacetylase (HDAC) inhibitors or demethylating agents, which should be explored in clinical studies.

Another promising pathway for targeted therapies is the JAK/STAT pathway with frequent *JAK3* mutations (13%). This rate is higher than the reported frequency in pediatric ALL patients[21]. For *JAK1*, varying mutation rates (4-18%) have been published[24,55]; in our cohort we found 4% of *JAK1* mutations. In total, 18% of all our adult T-ALL patients carried alterations in the JAK/STAT pathway, predominantly in high-risk patients with an immature immunophenotype that might benefit from the application of molecular directed therapies, including JAK inhibitors[56].

Interestingly, mutations in the spliceosome, which are frequently found in MDS and in subgroups of AML[52,53], were virtually absent in T-ALL patients throughout all subgroups. Therefore, other elementary cellular processes might play a role in T-ALL. In a recent study, alterations in posttranslational mechanisms were suggested[25]. Unfortunately, these findings were reported after the design of our study and genes of interest, like *RPL5*, *RPL10*, or *CNOT3*, were not included in our gene panel.

In contrast to the work of de Keersmaecker and colleagues, we did not observe an age dependent distribution of mutation frequencies[25]. However this is likely due to our study design focused on candidate genes potentially enriched for driver mutations. The difference in the mutation frequencies in unbiased whole exome approaches and the frequencies in selected gene panels raises the question, whether the higher rate of mutations in prima vista not T-ALL associated genes simply reflects the altered hematopoiesis in elderly or possesses itself a leukemogenic potential[57-59].

NGS techniques are becoming widely available and are about to guide treatment decisions. This offers the opportunity not only to identify targets but also to unravel the spectrum of subclonal architecture that likely affects the response to targeted therapies. In addition, the mutational spectrum of leukemic cell changes during the progression of the disease and relapses are frequently harbored in preexisting subclones[47,57]. It has been shown that specific mutations, which are only present in a minor subclone at diagnosis, could lead to relapse due to chemotherapy resistance[60,61]. Therefore, the sole assessment of mutated genes might insufficient to select an optimal targeted therapy and determination of mutation frequency might be necessary to predict responses and the risk of relapse. This additional level of complexity in describing mutational landscapes for each individual patient is explored in our study and emphasizes that reported drivers not only occurred in the founding clone, but also in subclones. Thus, the sole restriction to gene panel assays for diagnostic purposes will likely not be sufficient to capture the wide clonal diversity and thus will likely miss mutations in the founding clone and even more in the subclones[47].

Adult T-ALL reveals a highly heterogeneous and individual spectrum of candidate gene mutations. Here, we provide an original and comprehensive overview of recurring mutations that unravel altered pathways enriched in specific leukemic subgroups. In addition, we identified novel candidate genes with potential therapeutic implications (*FAT1*, *MLL2*, *HERC1*). These mutations have to be further validated in larger patient cohorts accompanied by functional assays regarding their value as potential therapeutic targets. The identification of individual lesions in daily clinical routine, their clonal evolution, and the incorporation of highly individualized therapies in study trials will be a future challenge.

## PATIENTS AND METHODS

### Patients and treatment

We investigated bone marrow samples from 81 adult T-ALL patients with material sent to the reference laboratory of the GMALL study group and with sufficient genomic DNA quality, quantity and blast count (>80%) to perform NGS (Supplementary Table S4). Immunophenotyping of fresh samples was centrally performed in the GMALL reference laboratory at the Charité, University Hospital Berlin, Germany. Immunophenotyping was carried out as previously described[62,63]. T-lineage leukemia was subclassified into pre-T-ALL or early T-ALL (cyCD3+, CD7+, CD5+/−, CD2−, sCD3−, CD4−/+, CD8−/+, CD1a− or cyCD3+, CD7+, CD5−, CD2+, sCD3−, CD4−, CD8−, CD1a−), thymic T-ALL (cyCD3+, CD7+, CD5+/−, CD2+/−, sCD3+/−, CD4+, CD8+, CD1a+), and mature T-ALL (cyCD3+, CD7+, CD5+, CD2+, sCD3+/−, CD4+/−, CD8+/−, CD1a−). The immature subtypes of pre-T-ALL and early T-ALL are merged to a combined early T-ALL group. In addition, patients were classified to have an ETP-ALL within early T-ALL according to the criteria originally used by Couston-Smith and colleagues[36]. In detail, all samples were positive for cyCD3 and CD7, with absence of CD1a and CD8 (less than 5% of all lymphoblasts were positive), and weak expression of CD5 (i.e. less than 75% of all lymphoblasts were positive). Furthermore, the immunophenotype was characterized by the expression (i.e. more than 25% of the lymphoblasts positive) of at least one myeloid or stem cell marker (CD13, CD33, CD65, CD117, CD34, HLA-DR).

**Table 2:** Characteristics of the investigated adult T-ALL patients Abbreviations: WBC, white blood cell count; TCR, T cell receptor

Number of patients		81
Sex	male	67
	female	14
Age	Median	35
	Range	17-73
WBC (/nl)	Median	41.9
	Range	0.8-332
Mediastinal mass (n=62)	yes	42
	no	20
TCR rearrangement (n=75)	yes	53
	no	22
Immunophenotype	thymic	40
	mature	15
	early	26

### Patients' characteristics

Of the 81 adult T-ALL patients examined in this study, 40 patients showed an immunophenotype of thymic T-ALL, 15 of mature T-ALL and 26 of early T-ALL, and amongst the latter group, 20 had an ETP-ALL immunophenotype. The median age was 35 years (range 17-73) and 83% of the patients were male. The median white blood cell count (WBC) at diagnosis was 41.9/nL (range 0.8-332; Table 2). For all 81 samples the TCR rearrangement status was available[64].

### Selection of candidate genes

The 88 genes selected for targeting sequencing are listed in the supplement (Supplementary Table S2). We selected genes known to be recurrently mutated in T-ALL, but also other genes, frequently mutated in BCP-ALL, (AML), and (MDS) were incorporated into the analysis[21,25,52,65]. Furthermore, genes with functions in epigenetic regulations, like members of the (PRC2) or in the splicing machinery, were included. Also, we selected candidate genes, based on our analysis of five exomes of adult ETP-ALL[22]. The targeted region of the 88 genes covered 1427 coding exons and 311 Kb of sequence (Supplementary Table S2).

### Targeted sequencing of candidate genes

We constructed libraries from 3 μg of genomic DNA, which were labeled by barcode indices (length: 6 bp). Customized biotinylated RNA oligo pools (SureSelect, Agilent) were used to hybridize the target regions comprising the 88 selected genes. We performed 76-bp paired-end sequencing on an Illumina Genome Analyzer IIx platform. Reads were mapped to NCBI hg19 RefSeq. For a variant call, we required at least a read depth of 30, an allele frequency of 20% and an average base calling quality of Q13. Polymorphisms annotated in dbSNP 135 were excluded (Supplementary Figure S2).

### Statistical analyses

Differences in the clinical characteristics were tested by the Pearson χ^2^ test resp. Fisher test. Differences in the mutation rate were analyzed by the Pearson χ^2^ test. For all tests a P-value < 0.05 (two-sided) was considered to indicate a significant difference. All calculations were performed using the SPSS software version 17 (SPSS Inc., Chicago, IL, USA) and GraphPad Prism® software version 5 (GraphPad Software Inc., La Jolla, CA, USA).

## SUPPLEMENTARY INFORMATION TABLES AND FIGURES


