# Supplementary material for: Mutational spectrum of adult T-ALL
Source: Oncotarget. 2014 Jul 15;6(5):2754–66. doi: 10.18632/oncotarget.2218 (PMC4413615; doi:10.18632/oncotarget.2218)
Supplement: Supplementary file 1 [file oncotarget-06-2754-s001.pdf]

Mutational spectrum of adult T-ALL

Supplementary Information

Supplementary Table S1: Immunophenotype of adult T-ALL patients.

(Numbers are percentage of positive cells)

ID	CD7	CD5	CD2	CD1	CYCD3	CD3	CD4	CD8	CD33	CD13	CD34	CD117
TE1	96	29	3	0	76	3	2	3	77	3	88	4
TE2	96	97	71	66	94	89	25	80	1	1	1	1
TE3	96	96	99	25	97	93	42	41	0	1	0	0
TE4	98	98	98	65	35	7	64	97	2	2	12	0
TE5	97	98	98	93	93	81	95	95	7	4	2	1
TE6	98	99	97	98	95	1	97	97	0	1	0	0
TE7	98	98	9	71	98	97	83	92	2	1	93	0
TE8	94	94	78	9	98	82	7	72	1	33	10	12
TE9	98	92	92	94	87	1	17	96	0	1	0	0
TE10	94	94	95	77	93	13	83	84	2	2	0	0
TE11	96	96	95	91	76	8	90	2	1	4	2	1
TE12	98	94	11	59	99	52	2	16	0	7	4	0
TE13	95	81	40	20	96	10	12	8	39	30	46	1
TE14	99	98	98	4	98	73	5	49	10	1	62	0
TE15	97	96	97	88	94	95	70	89	0	3	0	0
TE16	96	96	93	3	72	19	3	21	4	9	7	1
TE17	89	99	99	10	97	84	66	81	1	1	52	0
TE18	96	99	99	96	99	79	86	99	0	1	0	0
TE19	98	96	97	82	98	78	83	95	3	7	7	1
TE20	90	4	98	0	40	5	3	2	1	66	59	80
TE21	98	99	50	99	96	1	39	97	0	1	0	0
TE22	98	98	98	97	82	3	88	98	1	1	0	0
TE23	95	99	97	85	97	2	67	86	0	1	1	0
TE24	97	99	97	60	91	83	67	87	1	1	90	0
TE25	98	99	97	98	94	1	91	93	0	1	0	0
TE26	92	95	98	77	95	3	68	94	2	3	1	0
TE27	94	93	92	80	59	14	88	58	2	3	1	0
TE28	75	93	79	90	86	50	69	30	1	1	0	0
TE29	87	86	88	78	97	8	60	87	7	8	1	1
TE30	99	98	98	4	98	73	5	49	1	10	62	0
TE31	89	99	1	43	95	0	4	15	2	45	9	9
TE32	91	85	5	1	85	4	4	4	6	3	0	0
TE33	93	29	84	1	86	2	2	2	10	2	16	1
TE34	97	90	96	41	80	3	38	92	1	1	1	0
TE35	89	2	89	80	88	13	84	85	3	4	12	1
TE36	96	94	99	95	92	88	23	96	1	1	0	0
TE37	94	86	98	39	98	22	18	82	2	4	1	0
TE38	96	52	60	3	97	24	5	51	32	16	11	10
TE39	98	90	55	1	80	2	1	58	2	82	70	1
TE40	95	95	4	90	85	42	92	87	0	1	1	5
TE41	99	99	20	98	97	92	55	79	8	1	0	1
TE42	88	3	95	0	88	3	1	1	16	56	57	74
TE43	99	4	1	0	88	1	1	1	95	16	97	44
TE44	89	93	33	4	81	14	10	21	93	13	79	4
TE45	99	98	97	30	93	8	64	90	2	1	57	0
TE46	90	86	26	0	86	27	14	16	54	2	3	0
TE47	95	95	7	72	92	4	94	78	3	3	2	68
TE48	86	89	81	34	61	5	38	72	12	13	6	3
TE49	98	97	99	10	97	59	5	66	0	40	52	0
TE50	96	95	97	50	78	39	56	91	1	2	0	0
TE51	96	30	6	0	47	5	6	9	89	1	2	47
TE52	91	85	24	34	87	10	15	16	2	3	23	1
TE53	95	15	6	0	76	6	4	3	39	4	76	0
TE54	89	73	91	11	79	76	33	64	11	39	3	7
TE55	95	78	93	83	95	1	28	95	0	0	0	0
TE56	91	97	91	97	93	2	24	18	0	1	0	0
TE57	82	58	94	25	84	10	3	52	2	1	0	0
TE58	91	83	2	0	82	1	1	1	9	1	0	0
TE59	96	15	2	1	52	0	0	0	1	0	13	0
TE60	96	97	99	88	98	78	84	95	1	1	1	0
TE61	97	6	0	0	88	75	1	1	3	0	61	0
TE62	96	94	96	43	68	13	89	71	2	3	1	1
TE63	85	92	5	1	81	5	3	57	53	55	46	1
TE64	97	97	93	94	95	11	22	96	4	6	32	2
TE65	94	84	93	1	90	11	6	8	12	56	11	41
TE66	98	24	4	4	88	4	2	2	3	48	1	4
TE67	87	36	74	3	94	69	71	3	3	1	2	0
TE68	71	2	19	1	48	2	2	2	8	47	82	42
TE69	99	49	81	0	93	2	2	3	44	0	82	3
TE70	82	3	87	1	87	1	1	1	1	31	52	85
TE71	74	3	5	0	95	4	4	2	95	53	92	62
TE72	95	5	3	1	78	3	2	2	0	71	90	89
TE73	98	66	4	0	82	3	2	3	80	0	68	1
TE74	97	6	0	0	88	75	1	1	3	0	61	0
TE75	98	28	3	0	70	3	2	0	95	0	22	0
TE76	89	2	93	1	83	2	1	1	3	27	14	44
TE77	93	9	3	2	69	4	5	3	5	5	3	42
TE78	97	42	99	1	84	4	3	2	9	68	0	3
TE79	91	12	31	4	85	6	3	4	7	52	55	1
TE80	72	6	71	0	34	3	2	1	0	95	91	0
TE81	74	13	90	0	54	4	4	2	15	20	96	63

Supplementary Table S2: Selected genes according to their annotated pathway. Selection is described in Materials and Methods.

Pathway	Gene (sequence length in bp)
**DNA methylation**	DNMT3A (4380), TET2 (9233), IDH1 (2382), IDH2 (2694)
**DNA repair**	MSH2 (3307), FRAP1 (8677), HERC1 (15196), PRKCZ (2326), PIK3C2B (7686)
**JAK/STAT pathway**	IL-7RA (4626), JAK1 (5047), JAK2 (5285), JAK3 (3996)
**MAPK signaling**	NRAS (4449), KRAS (5765), TP53 (2579), BRAF (2480)
**Methyltransferase**	MLL2 (19419), WHSC1 (8568)
**NOTCH signaling**	NOTCH1 (9371), FBXW7 (3734), NOTCH2 (11389), NOTCH3 (8070), NOTCH4 (6745), DLL1 (3174), DLL3 (2380), DLL4 (3383), CREBBP (10798), JAG1 (5901), JAG2 (5835), HES1 (1578), DTX1 (3455)
**PIK3A/AKT pathway**	AKT1 (2866), AKT2 (5300), AKT3 (5127), PIK3CA (9093), PIK3C2B (7686)
**PRC**	BMI1 (3428), EZH2 (2466), EP300 (9585), SUZ12 (4472), EED (2223)
**Tyrosine kinases**	FLT3 (3842), KIT (5186), ABL1 (5766)
**Spliceosome**	U2AF35 (962), ZRSR2 (1491), SRSF2 (2845), SF3A1 (3510), SF3B1 (4336), PRPF40B (3154), U2AF65 (3130), SF1 (3094)
**Transcription factor**	MEF2C (4079), BCL11B (7559), RUNX1 (7274), WT1 (3122), ERG (4919), GATA1 (1497), GATA2 (3367), GATA3 (3078), ETV6 (2211), DNM2 (3654)
**WNT pathway**	FAT1 (14786), FAT3 (18474), LEF1 (3068)
**Others**	PTEN (9027), CDKN2A (12218), ASXL1 (7031), CB2 (5254), CEBPa (2631), KDM6A (5438), CBL (10257), BAALC (2831), IGFBP7 (1427), MYC (2345), ICAM-1 (3246), DICER1 (10277), PHF6 (4489), IKZF1 (6189), CTNNA3 (3232), SH2B3 (5406), GFI1 (2855), BCOR (6358), RELN (11571), ECT2L (4319), HNRNPA1 (4121), ERAP2 (5705)

Supplementary Table S3: Sequencing results for each patient.

	# of total reads:	# of sequenced bases mapped to genome:	% of bases mapped in HQ reads:	% of target sequenced:	% of target sequenced min 10x coverage:	% of target sequenced min 20x coverage:	mean target coverage:	% bases on or near target:	# of mutations
**TE1**	2,045,753	159,298,509	98.8	93.3	82.7	80.0	131.3	31.7	4
**TE2**	1,832,324	139,967,208	98.1	93.5	82.6	79.3	117.9	33.9	5
**TE3**	894,884	64,715,854	96.1	93.0	77.0	65.8	42.4	26.1	4
**TE4**	431,948	31,860,464	97.0	92.9	75.7	64.6	35.3	42.8	22
**TE5**	557,987	41,024,608	96.9	93.0	77.8	67.4	40.6	38.7	31
**TE6**	684,663	51,865,125	97.8	93.0	79.9	74.9	63.7	45.6	20
**TE7**	1,449,898	111,590,905	98.8	93.6	83.5	81.5	144.2	51.0	3
**TE8**	1,255,877	95,713,588	98.8	93.6	83.9	82.0	130.8	57.8	2
**TE9**	1,427,375	109,859,986	99.0	93.7	83.9	82.2	155.5	58.4	3
**TE10**	2,039,327	158,201,114	99.0	93.8	84.4	83.2	227.2	56.1	2
**TE11**	1,203,429	90,947,520	98.7	93.6	83.7	81.5	124.0	58.9	4
**TE12**	1,034,614	75,365,488	97.4	93.4	82.9	79.5	87.9	49.0	6
**TE13**	1,156,668	83,413,871	97.4	93.3	83.0	79.9	97.9	49.1	2
**TE14**	925,543	69,363,991	98.3	93.1	81.7	78.6	89.1	49.8	3
**TE15**	1,714,059	132,689,620	99.0	93.5	83.7	82.2	190.5	56.2	5
**TE16**	1,509,019	115,965,391	99.0	93.4	83.6	81.7	153.9	53.3	2
**TE17**	1,288,221	96,650,746	98.5	93.3	83.1	81.1	130.9	53.9	3
**TE18**	1,668,937	129,149,002	99.0	93.6	83.6	82.1	186.2	56.0	6
**TE19**	1,175,362	87,662,622	98.7	93.5	82.4	79.8	108.5	54.6	8
**TE20**	2,102,967	158,288,170	98.1	93.6	83.9	82.5	214.1	58.1	3
**TE21**	775,628	57,936,692	97.8	93.0	79.7	70.0	72.6	53.3	3
**TE22**	1,379,518	105,875,229	98.3	93.3	82.7	81.2	121.3	47.4	3
**TE23**	1,742,516	135,314,096	99.0	93.7	84.0	82.6	165.8	48.7	5
**TE24**	892,434	66,227,168	98.1	93.5	82.5	79.1	66.1	46.8	5
**TE25**	2,180,384	166,875,004	98.4	93.4	83.1	81.9	190.8	48.0	10
**TE26**	973,722	71,650,660	96.7	93.2	81.4	76.5	64.3	38.8	3
**TE27**	1,112,210	82,016,677	97.9	93.5	83.4	81.0	89.4	50.1	2
**TE28**	882,373	64,365,539	97.8	93.4	82.3	79.1	74.3	52.9	2
**TE29**	852,041	62,647,512	97.8	93.2	81.8	77.0	87.5	55.6	8
**TE30**	1,343,011	98,975,171	97.5	93.5	83.8	81.9	118.1	51.2	5
**TE31**	1,034,327	75,849,270	98.0	93.4	82.9	80.2	88.6	53.7	1
**TE32**	1,213,604	90,791,805	98.2	93.2	83.8	82.0	128.0	57.3	4
**TE33**	1,713,217	126,501,751	97.7	93.8	84.0	82.4	155.2	51.1	6
**TE34**	896,772	64,787,634	97.3	93.3	82.4	79.2	79.7	52.3	2
**TE35**	1,675,609	125,989,726	97.5	93.9	84.2	82.5	107.1	38.5	8
**TE36**	1,697,055	125,254,162	98.0	93.4	83.8	82.1	169.0	57.0	4
**TE37**	1,096,139	78,795,131	96.6	93.2	81.2	76.5	79.8	44.3	2
**TE38**	1,403,658	102,668,960	97.9	93.5	83.8	82.0	134.5	52.7	4
**TE39**	1,160,904	84,122,948	97.8	93.5	83.3	80.8	105.4	51.6	8
**TE40**	1,036,558	75,371,150	97.8	93.2	82.2	79.5	94.3	55.4	4
**TE41**	767,433	57,253,762	98.5	93.5	81.5	77.2	63.4	48.9	8
**TE42**	1,312,643	97,920,658	98.5	93.3	83.4	81.5	144.3	58.2	3
**TE43**	1,349,752	101,408,187	98.5	93.5	82.9	80.9	142.8	54.7	1
**TE44**	1,408,447	106,803,166	98.8	93.4	83.7	81.7	169.2	60.8	5
**TE45**	887,904	63,864,706	97.0	93.2	80.6	76.2	72.9	48.2	2
**TE46**	1,145,051	84,329,972	98.0	93.5	82.4	79.4	91.1	43.0	6
**TE47**	809,493	59,086,770	97.8	93.2	81.1	75.5	70.1	49.0	4
**TE48**	1,279,162	93,025,958	96.8	93.4	82.4	79.2	83.6	38.4	2
**TE49**	839,676	60,356,033	98.2	93.0	79.6	74.2	63.3	44.5	4
**TE50**	1,227,260	90,708,396	98.5	93.7	82.4	80.0	95.6	45.3	4
**TE51**	872,686	62,510,041	98.5	93.2	79.0	72.8	70.2	48.3	1
**TE52**	1,338,833	100,489,669	97.0	93.6	82.6	78.6	75.7	32.3	7
**TE53**	972,261	72,175,042	97.6	93.5	82.1	78.1	65.7	42.5	2
**TE54**	1,921,389	142,176,047	97.9	93.7	83.9	82.3	142.4	43.5	4
**TE55**	706,567	52,080,425	96.9	93.2	80.2	70.5	52.0	42.3	4
**TE56**	1,322,526	99,566,801	98.5	93.5	83.3	79.3	132.8	56.8	4
**TE57**	1,009,170	72,470,031	96.1	93.2	80.6	74.1	60.2	35.3	2
**TE58**	1,207,946	91,745,654	98.9	93.5	84.1	82.1	149.3	67.7	9
**TE59**	1,018,383	75,209,252	98.2	93.5	83.1	78.8	106.1	62.6	8
**TE60**	645,529	49,434,468	99.0	93.2	81.6	71.4	78.3	66.0	7
**TE61**	448,886	34,342,922	98.7	93.1	79.4	65.4	50.8	60.7	5
**TE62**	1,030,986	79,313,634	99.4	93.7	84.5	83.1	133.7	73.1	4
**TE63**	1,283,251	99,450,751	99.4	93.3	84.2	82.7	168.8	68.6	3
**TE64**	2,427,564	188,860,380	99.5	93.5	84.8	83.8	330.3	69.6	4
**TE65**	609,015	46,798,734	99.4	93.2	82.5	79.6	87.2	75.8	4
**TE66**	811,097	62,297,334	99.5	93.5	83.8	82.0	118.1	78.8	3
**TE67**	579,487	44,093,889	99.3	93.2	80.9	70.3	86.4	79.8	6
**TE68**	759,765	55,696,015	98.7	93.3	83.6	81.1	91.7	70.3	3
**TE69**	932,698	68,412,234	98.5	93.4	83.7	81.5	104.2	64.9	4
**TE70**	734,833	54,103,729	98.8	93.5	83.6	81.1	90.5	74.1	8
**TE71**	890,031	64,673,202	98.6	93.3	81.6	77.7	90.3	67.0	5
**TE72**	1,481,825	110,076,129	98.2	93.5	84.6	83.1	155.9	59.8	4
**TE73**	966,305	71,310,575	98.5	93.3	83.8	81.8	114.7	66.9	4
**TE74**	1,141,746	88,019,727	99.4	93.4	84.3	82.6	163.0	76.4	6
**TE75**	625,161	45,713,633	98.3	93.3	81.6	76.7	62.9	63.2	6
**TE76**	696,727	50,803,562	98.7	93.1	82.4	79.3	88.0	68.2	5
**TE77**	1,558,686	116,636,060	98.3	93.4	84.2	82.8	163.6	57.8	3
**TE78**	963,936	73,472,392	99.3	93.2	83.5	80.9	142.6	77.0	8
**TE79**	2,146,597	166,617,273	99.3	93.4	84.1	82.8	252.0	57.8	7
**TE80**	2,768,317	203,188,308	98.1	93.6	84.6	83.4	249.1	49.8	2
**TE81**	6,139,294	480,817,719	99.3	94.2	85.5	84.9	641.5	53.7	5
**Median**	1,145,051	84,122,948	98.3	93.4	83.1	80.8	106.1	53.7	4

Supplementary Table S4: All SNVs found in 96 T-ALL see file Supplementary Tables S4

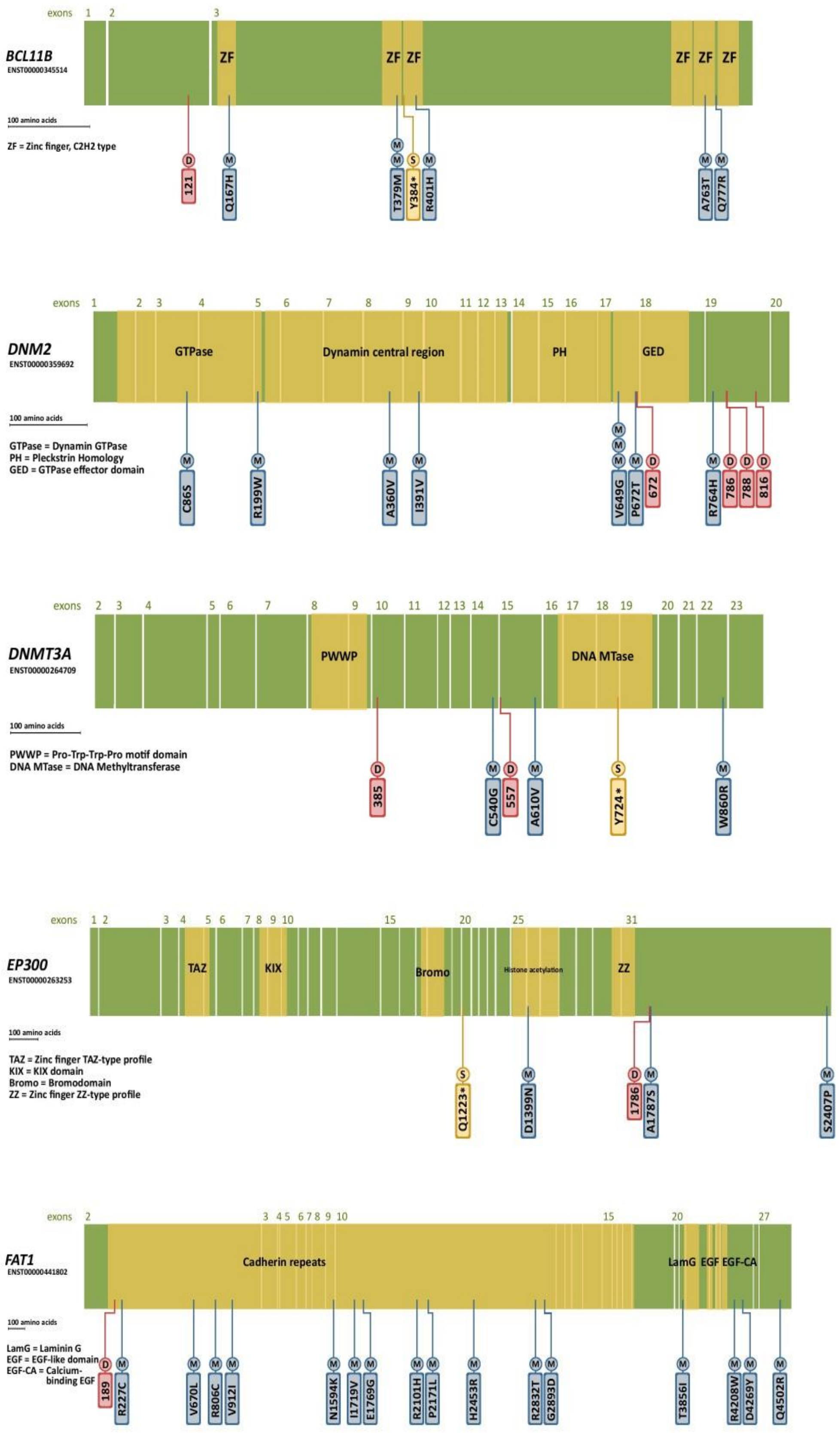
exons 1 2 3
BCL11B
ENST00000345514
ZF ZF ZF ZF ZF ZF
100 amino acids
ZF = Zinc finger, C2H2 type
D 121
M Q167H
M M T379M
S Y384*
M R401H
M A763T
M Q777R
exons 1 2 3 4 5 6 7 8 9 10 11 12 13 14 15 16 17 18 19 20
DNM2
ENST00000359692
GTPase
Dynamin central region
PH
GED
100 amino acids
GTPase = Dynamin GTPase
PH = Pleckstrin Homology
GED = GTPase effector domain
M C86S
M R199W
M A360V
M I391V
M M M V649G
M P672T
D 672
M R764H
D 786
D 788
D 816
exons 2 3 4 5 6 7 8 9 10 11 12 13 14 15 16 17 18 19 20 21 22 23
DNMT3A
ENST00000264709
PWWP
DNA MTase
100 amino acids
PWWP = Pro-Trp-Trp-Pro motif domain
DNA MTase = DNA Methyltransferase
D 385
M C540G
D 557
M A610V
S Y724*
M W860R
exons 1 2 3 4 5 6 7 8 9 10 15 20 25 31
EP300
ENST00000263253
TAZ
KIX
Bromo
Histone acetylation
ZZ
100 amino acids
TAZ = Zinc finger TAZ-type profile
KIX = KIX domain
Bromo = Bromodomain
ZZ = Zinc finger ZZ-type profile
S Q1223*
M D1399N
D 1786
M A1787S
M S2407P
exons 2 3 4 5 6 7 8 9 10 15 20 27
FAT1
ENST00000441802
Cadherin repeats
LamG EGF EGF-CA
100 amino acids
LamG = Laminin G
EGF = EGF-like domain
EGF-CA = Calcium-binding EGF
D 189
M R227C
M V670L
M R806C
M V912I
M N1594K
M I1719V
M E1769G
M R2101H
M P2171L
M H2453R
M R2832T
M G2893D
M T3856I
M R4208W
M D4269Y
M Q4502R

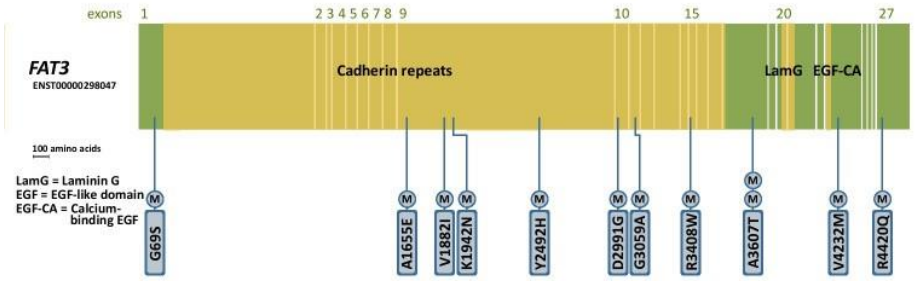
exons 1 2 3 4 5 6 7 8 9 10 15 20 27
FAT3
ENST00000298047
Cadherin repeats
LamG EGF-CA
100 amino acids
LamG = Laminin G
EGF = EGF-like domain
EGF-CA = Calcium-binding EGF
G69S
A1655E
V1882I
K1942N
Y2492H
D2991G
G3059A
R3408W
A3607T
V4232M
R4420Q

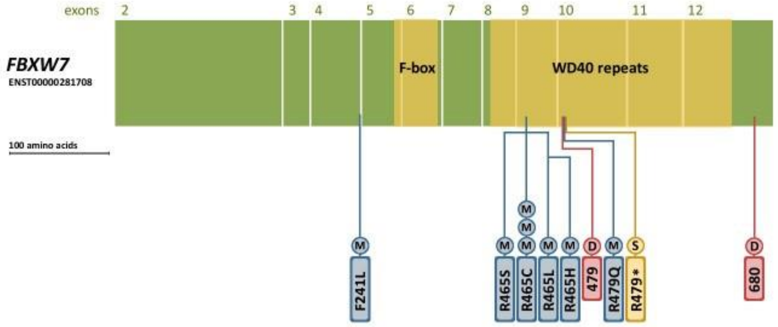
exons 2 3 4 5 6 7 8 9 10 11 12
FBXW7
ENST00000281708
F-box
WD40 repeats
100 amino acids
F241L
R465S
R465C
R465L
R465H
479
R479Q
R479*
680

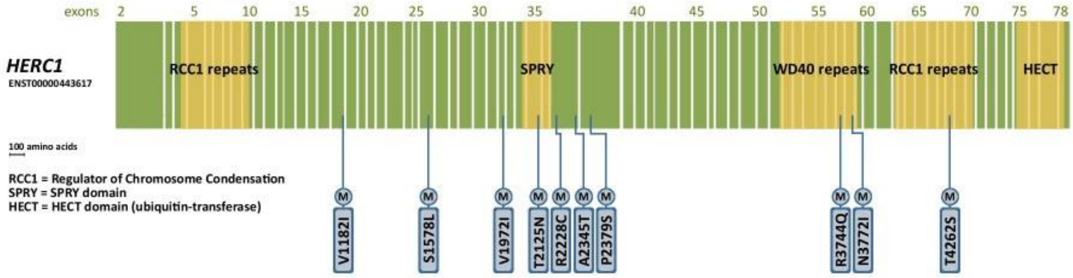
exons 2 5 10 15 20 25 30 35 40 45 50 55 60 65 70 75 78
HERC1
ENST00000443617
RCC1 repeats
SPRY
WD40 repeats
RCC1 repeats
HECT
100 amino acids
RCC1 = Regulator of Chromosome Condensation
SPRY = SPRY domain
HECT = HECT domain (ubiquitin-transferase)
V1182I
S1578L
V1972I
T2125N
R2228C
A2345T
P2379S
R3744Q
N3772I
T4262S

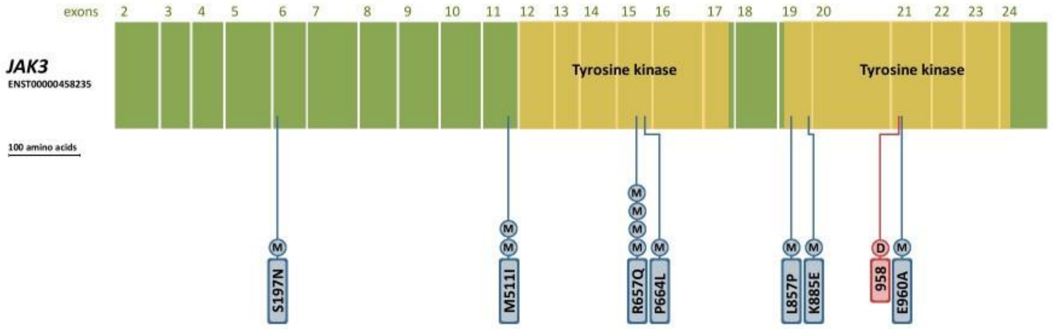
exons 2 3 4 5 6 7 8 9 10 11 12 13 14 15 16 17 18 19 20 21 22 23 24
JAK3
ENST00000458235
Tyrosine kinase
Tyrosine kinase
100 amino acids
S197N
M511I
R657Q
P664L
L857P
K885E
958
E960A

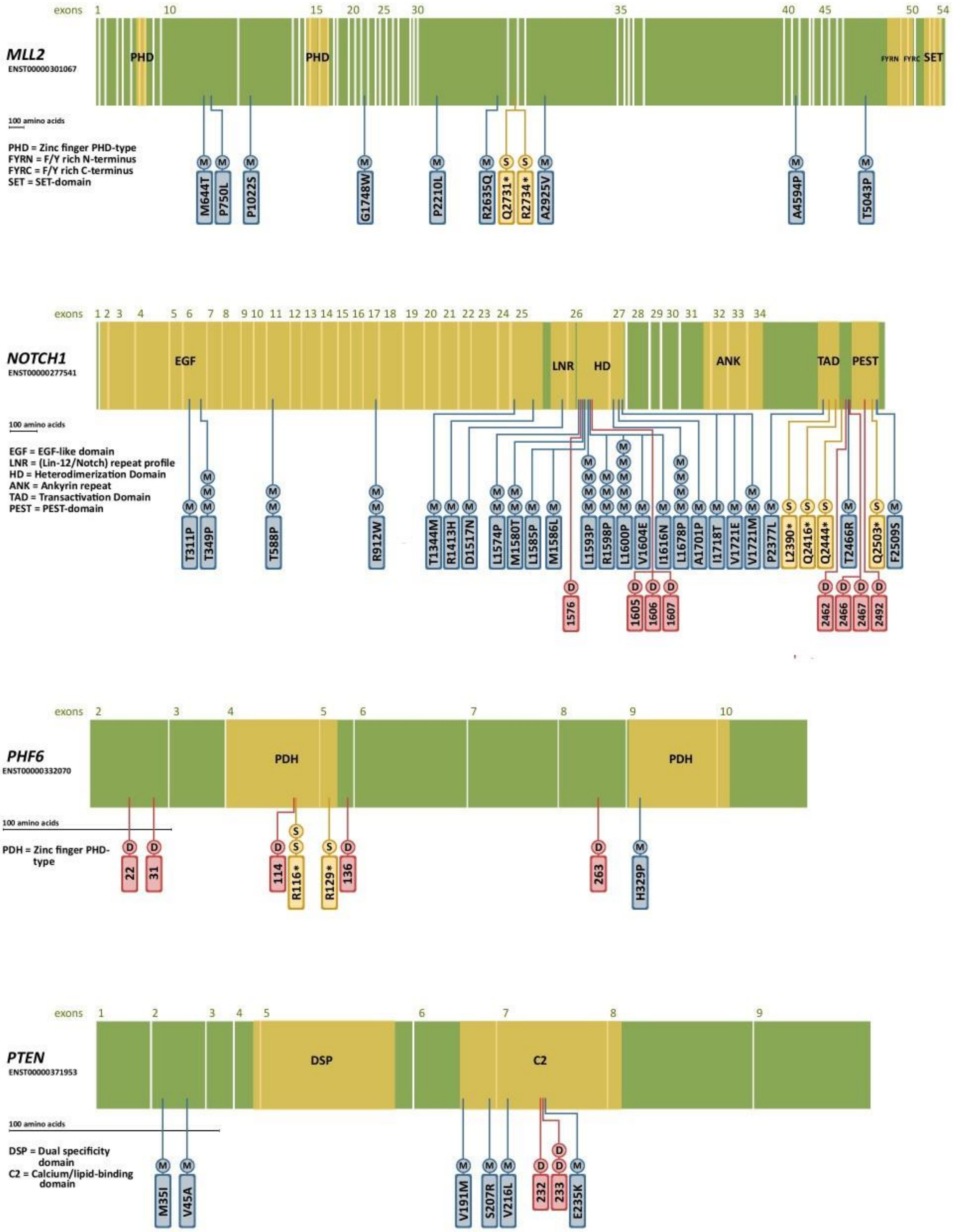

exons 1 10 15 20 25 30 35 40 45 50 54
MLL2
ENST00000301067
PHD
PHD
FYRN FYRC SET
100 amino acids
PHD = Zinc finger PHD-type
FYRN = F/Y rich N-terminus
FYRC = F/Y rich C-terminus
SET = SET-domain
M644T P750L P1022S G1748W P2210L R2635Q Q2731* R2734* A2925V A4594P T5043P
exons 1 2 3 4 5 6 7 8 9 10 11 12 13 14 15 16 17 18 19 20 21 22 23 24 25 26 27 28 29 30 31 32 33 34
NOTCH1
ENST00000277541
EGF
LNR
HD
ANK
TAD
PEST
100 amino acids
EGF = EGF-like domain
LNR = (Lin-12/Notch) repeat profile
HD = Heterodimerization Domain
ANK = Ankyrin repeat
TAD = Transactivation Domain
PEST = PEST-domain
T311P T349P T588P R912W T1344M R1413H D1517N L1574P M1580T L1585P M1586L L1593P R1598P L1600P V1604E I1616N L1678P A1701P I1718T V1721E V1721M P2377L L2390* Q2416* Q2444* T2466R Q2503* F2509S
1576 1605 1606 1607 2462 2466 2467 2492
exons 2 3 4 5 6 7 8 9 10
PHF6
ENST00000332070
PDH
PDH
100 amino acids
PDH = Zinc finger PHD-type
22 31 114 R116* R129* 136 263 H329P
exons 1 2 3 4 5 6 7 8 9
PTEN
ENST00000371953
DSP
C2
100 amino acids
DSP = Dual specificity domain
C2 = Calcium/lipid-binding domain
M35I V45A V191M S207R V216L 232 233 E235K

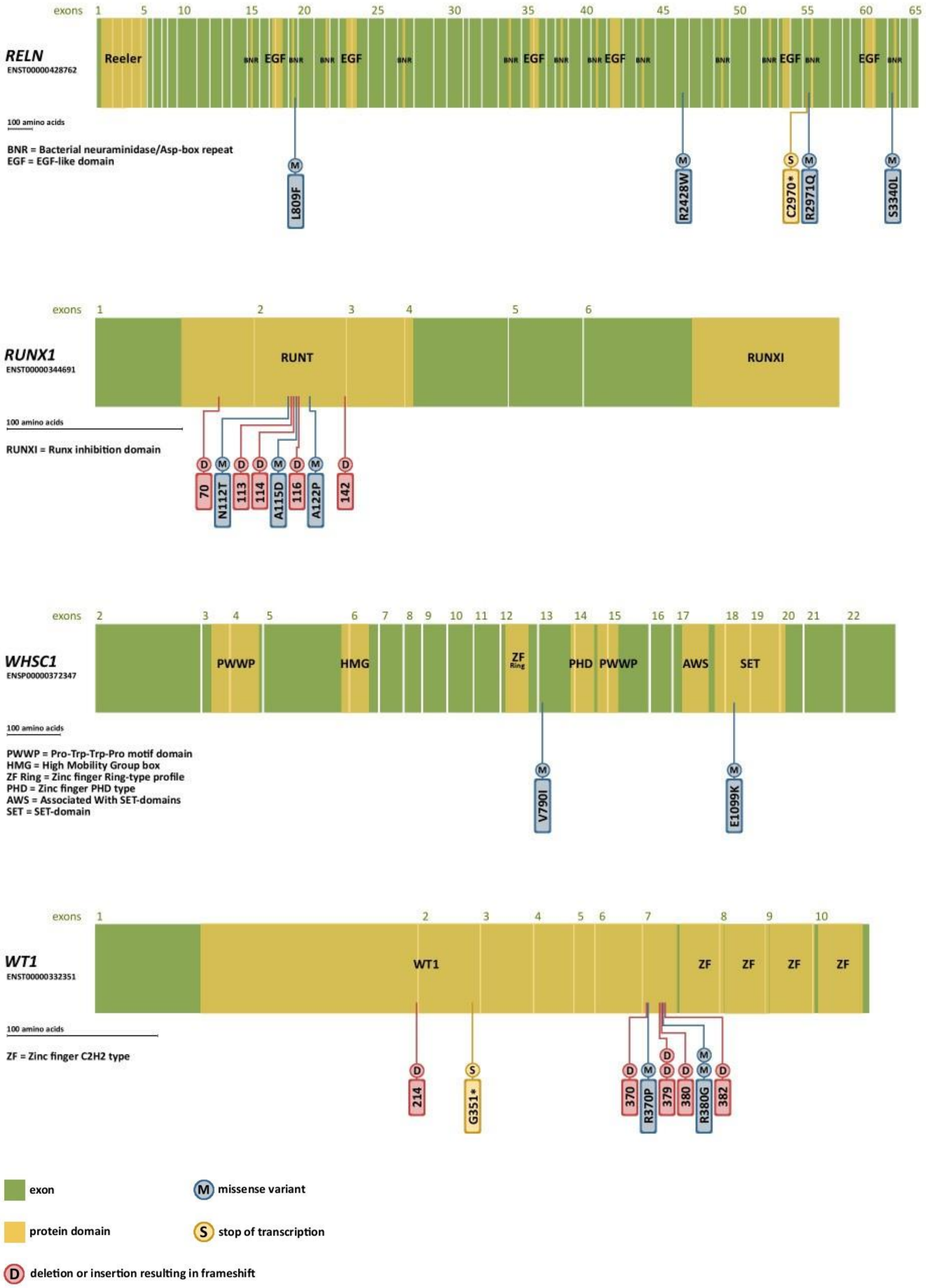

Supplementary Figure S1: Protein domain plots. Protein plots are shown for genes with at least five mutations in the 81 adult T-ALL patients.

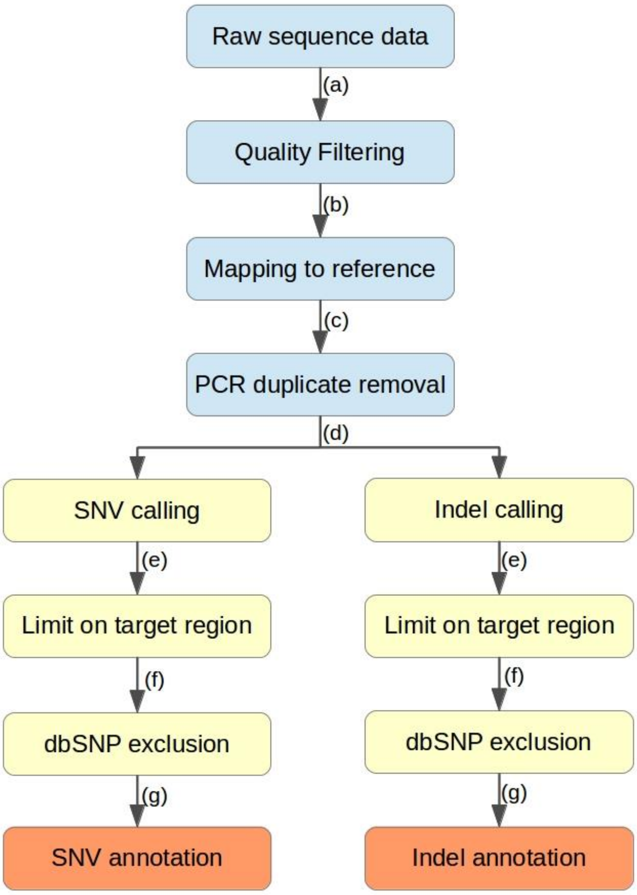

Supplementary Figure S2: Workflow to identify recurrent somatic mutations in T-ALL .

(a) Illumina chastity filter and read trimming, (b) read mapping to reference genome using BWA, (c) removing PCR duplicates using SAMtools, (d) variant calling using Varscan, (e) limit variants to target region, (f) remove known polymorphisms using dbSNP135, (g) annotate variants using SnpEff.

Abbreviations: SNV – single nucleotide variant; Indel – short insertion or deletion
